# Toward a taxonomy of trust for probabilistic machine learning

**DOI:** 10.1126/sciadv.abn3999

**Published:** 2023-02-15

**Authors:** Tamara Broderick, Andrew Gelman, Rachael Meager, Anna L. Smith, Tian Zheng

**Affiliations:** ^1^ Department of Electrical Engineering and Computer Science, Massachusetts Institute of Technology, Cambridge, MA, USA.; ^2^ Department of Statistics, Columbia University, New York, NY, USA.; ^3^ Department of Political Science, Columbia University, New York, NY, USA.; ^4^ Department of Economics, London School of Economics and Political Science, London, UK.; ^5^ Department of Statistics, University of Kentucky, Lexington, KY, USA.

## Abstract

Probabilistic machine learning increasingly informs critical decisions in medicine, economics, politics, and beyond. To aid the development of trust in these decisions, we develop a taxonomy delineating where trust in an analysis can break down: (i) in the translation of real-world goals to goals on a particular set of training data, (ii) in the translation of abstract goals on the training data to a concrete mathematical problem, (iii) in the use of an algorithm to solve the stated mathematical problem, and (iv) in the use of a particular code implementation of the chosen algorithm. We detail how trust can fail at each step and illustrate our taxonomy with two case studies. Finally, we describe a wide variety of methods that can be used to increase trust at each step of our taxonomy. The use of our taxonomy highlights not only steps where existing research work on trust tends to concentrate and but also steps where building trust is particularly challenging.

## Teaser

A taxonomy delineates where trust can break down in a probabilistic&nbsp;machine learning workflow that informs critical decisions.

“Science, at its core, is a social phenomenon. It is a reflection of people, of our relationships, and of our institutions. When we provide inputs to the algorithm, when we program the device, when we design, test, and research, we are making human choices—choices that bring our social world to bear in a new and powerful way.” — Alondra Nelson, Deputy Director for Science and Society, White House Office of Science and Technology Policy, 2021.

## INTRODUCTION

Machine learning (ML) in general, and probabilistic methods in particular, are increasingly used to make major decisions in science, the social sciences, and engineering, with the potential to profoundly affect individuals’ day-to-day lives. For instance, probabilistic methods have driven knowledge of the spread and effects of severe acute respiratory syndrome coronavirus 2 (SARS-CoV-2) (*1*–*3*), underlie election predictions at the *Economist* (*4*, *5*), and can guide our understanding of the efficacy of microloans in alleviating poverty (*6*). Given the large and growing impact of probabilistic ML, it behooves us to make sure that its outputs are useful for its users’ stated purposes.

There are many potential points, though, where a data analysis pipeline may break down. This issue becomes especially pressing as statistics and ML workflows grow increasingly complex to face modern challenges. These challenges arise not only from the sheer size of the data but also from the inherent difficulty of the problems being studied. "Big data" are messy data: confounded data rather than random samples, observational data rather than experiments, and available data rather than direct measurements of underlying constructs of interest. To make relevant inferences from big data, we need to extrapolate from sample to population, from control to treatment group, and from measurements to latent variables. All of these steps require modeling. In addition, big data need big models: latent-variable models for psychological states or political ideologies, differential equation models in pharmacology, dynamic image analysis, and so forth. To meet modern challenges, we often fit models that are on the edge of our ability to compute and interact with in the real world. These models typically contain many assumptions and decision points. Also, we are often pushed to adjust for more factors to capture the complexity of our world. However, models that adjust for lots of factors are hard to estimate. Fitting big models to big data requires scalability, so we must often turn to approximations such as Markov chain Monte Carlo, variational inference, and expectation propagation.

As this pipeline becomes more elaborate, there are more potential points of failure. But other complex endeavors succeed thanks, in large part, to extensive infrastructure: e.g., software engineering has testing and construction has scaffolding. It is similarly necessary to build an infrastructure to support the trustworthy creation and deployment of methods in probabilistic ML. To this end, there exists a large literature addressing concerns of trust in data science, with key words including reproducibility, replicability, theoretical guarantees, stability, and interpretability.

To a new reader, it may not be obvious how these concerns relate to each other and what new work is needed in this area. In the present paper, we develop a “taxonomy of trust,” splitting the probabilistic ML workflow (or data analysis pipeline or inferential chain) into distinct constituent parts where trust can fail. Just as testing in software engineering benefits from modularity, we hope that the modularity of our taxonomy can facilitate more targeted work on trust concerns. We will use our taxonomy to highlight concerns that are relatively well studied and those that could benefit from more attention.

We start by delineating our focus as it relates to probabilistic ML and trust. Then, we lay out our taxonomy and illustrate its parts with two case studies. Finally, we use our taxonomy to organize and discuss different approaches to growing trust in a data analysis, including existing work on reproducibility and replicability.

## OUR FOCUS

We here focus on probabilistic ML methods. Following the textbook *Machine Learning: a Probabilistic Perspective*, “we define machine learning as a set of methods that can automatically detect patterns in data, and then use the uncovered patterns to predict future data, or to perform other kinds of decision making under uncertainty” (*7*). Furthermore, in accordance with (*7*, *8*), we describe an ML method as probabilistic when it uses the tools of probability theory to handle uncertainty in the decision-making process, as in our examples.

In what follows, we concentrate on trust in the quality of decisions that are made using the results of a data analysis. We focus on the extent to which informed experts can trust these decisions; these experts could include the original data analysts—as well as peer data analysts in academia, industry, journalism, other forms of public service, or an independent capacity. Further stakeholders typically rely on experts to vouch for the products of a data analysis, so our trust focus remains relevant to these stakeholders as well.

Trust of lay consumers in a data analysis, though, may also rely on other factors. For instance, the broader sociopolitical climate can influence a broader sense (or lack) of trust in the scientific community and its outputs, but a discussion of these relationships is outside the scope of the present work. We point the interested reader to resources on human trust in artificial intelligence (AI) (*9*, *10*), trust in computing (*11*), and trust more generally (*12*). Likewise, concerns about privacy and security are important and related to trust in the use of data and its artifacts (*13*, *14*) but are outside our purview.

## WHERE TRUST CAN BREAK DOWN

**Fig. 1.**
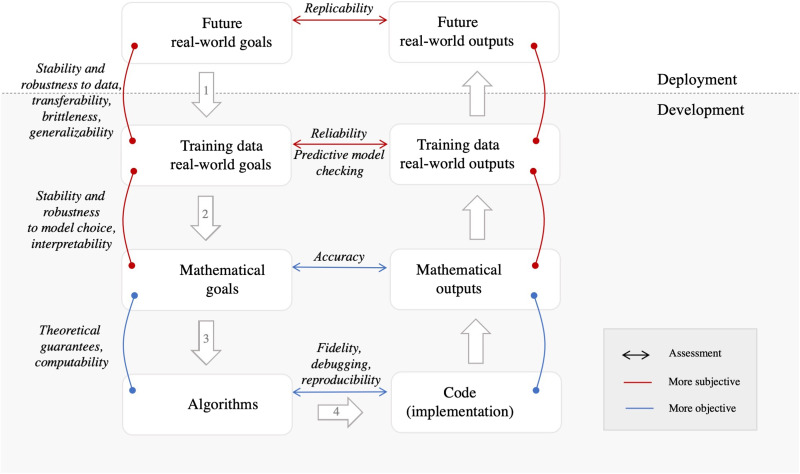
Diagram illustrating steps where trust can break down in a data analysis workflow. See the section “Where trust can break down” for a detailed description of the (nonitalicized) steps. See the section “Building trust” for a discussion of the italicized connectors, which include many of the characteristics of Schwartz *et al.* (*83*) for cultivating trust in AI systems.

We next turn to understanding where trust in the decisions made from a probabilistic ML analysis can fracture. Figure 1 gives a visualization of our taxonomy of trust; this graphic provides a map of how probabilistic ML analyses interact with the real world. We first give a high-level overview of its steps, then describe how it shows where trust can break down in a data analysis, and finally give two case studies as examples.

We typically embark upon a data analysis because we seek to make an impact on our understanding of the world or generate future decision recommendations. From this perspective, an analysis is designed with “future real-world goals” in mind (upper left of Fig. 1). The data analyst then follows a rough workflow (on the left side of the figure) to turn this abstract goal into a concrete set of actions, which correspond to the numbered steps (arrows) in Fig. 1.

1) To serve these real-world goals, data must be gathered and processed for analysis. At this point, the analyst has reduced the problem to “training-data real-world goals.”

2) The analyst chooses a model and expresses the real-world problem as a mathematical problem. The analyst has now reduced the problem to concrete “mathematical goals.”

3) The analyst chooses particular “algorithms” to solve the mathematical problem.

4) The analyst runs the algorithm in practice using some particular “code (implementation).”

We see the outputs of this workflow on the right side of Fig. 1. The analyst runs their code and obtains a particular mathematical output. The output on the training data at hand is interpreted in the context of the real-world goal. Finally, the learned model is applied to future real-world outputs to make substantive conclusions and decisions.

Each numbered step in Fig. 1 in turn represents a point where trust can break down in a data analysis.

1) Researchers typically face constraints in the cost of gathering, processing, or analyzing data, so the dataset is necessarily a limited representation of the world. In choosing to approach our abstract question with a particular analysis of a particular set of data, we trust that the results from our data analysis are relevant to conclusions and decisions we will make at other places or times.

2) Essentially, all models are misspecified. In choosing to express our problem with a model and mathematical formalism, we are trusting that the model and formalism adequately capture the substantive goals of the analysis.

3) Algorithms are often supported by theory but based on assumptions that may not be perfectly achieved in practice. In choosing to solve our mathematical problem with a particular algorithm, we are trusting that the algorithm accomplishes the particular mathematical goal.

4) Code is a precise way to manifest an algorithm, but it is difficult to avoid bugs and conceptual errors, especially in large and complex code bases. In choosing to execute our algorithm via code, we are trusting that the code is faithful to the algorithm.

### Case study: Microcredit efficacy

We use an example from applied economics that one of us has worked on (*6*) to demonstrate how the steps of Fig. 1 relate to trust in a data analysis.

#### Future real-world goals

Economists and policymakers would like to know if programs promoting microcredit (small loans in developing countries) have an overall beneficial effect—and, if they do, to use that information to inform decisions on subsidizing or disbursing microcredit across many potential locations. A related question of interest to economists is whether, in a given context, expanding access to the services offered by local microlenders would be beneficial for a local population.

#### Step 1: Future real-world goals to training-data real-world goals

We analyzed data from seven different microfinance programs, each located in a different country. The countries range from Mexico to Mongolia, Bosnia to the Philippines. The programs encompassed for-profit banks, government programs, and non-governmental organizations. Each individual dataset has been used to understand local efficacy of expanding access to microloans. In (*6*), we combined data across different settings and even somewhat different loan products to assess the geographically broader question of expanding microcredit in new locations.

In each of the individual studies, microcredit access was assigned in a randomized controlled trial (RCT) to prevent selection bias on the part of the microlender or borrowers from confounding the estimate of the effect of microcredit.

Moreover, researchers in each case decide what “beneficial” means, then what variables to measure to capture it, and further how to measure these variables using surveys or other tools. In the microcredit RCTs, the researchers all considered small-business profits as an outcome, but only five of the seven studies considered household consumption, and even fewer considered other components of well-being such as community health. All of these variables were carefully conceptualized and measured; e.g., on the basis of contextual knowledge and previous research, someone decides whether, in rural Mexico, goats count as consumption or as investment. Some of the studies also recorded contextual covariates or characteristics of households to understand how they affect microcredit efficacy, but the relative importance of different factors in driving household outcomes (and thus likely moderating the effects of credit) such as consumption or business profits is itself an open research question.

##### Trust

Trust could break down in this step if the particular data analysis proved not to be useful for making future decisions. A researcher almost always makes choices that trade off the practicality and feasibility of collecting data and running the analysis against accurately capturing the state of the world. On the data collection side, there is an unending set of outcomes and characteristics of homes or small businesses that researchers could collect, but for time and funding reasons, the list must usually be limited.

The preference for randomized trials of microcredit reflects a desire to estimate unconfounded treatment effects even without being able to do any covariate adjustment, which partially addresses this concern, but might at times result in greater noise or less representative sample sizes and thus greater extrapolation error.

Finally, the use of multiple studies provides hope that the results will generalize more than any single study, but full generalization relies on the assumption that the studies we have cover relevant aspects of a broader population of contexts in which we might make policy. And even if this assumption is satisfied, all studies occur in the past, but the policy decision gets made in the future; it is necessary to trust that the inference remains applicable under the new circumstances or to make assumptions allowing appropriate adjustments to make this extrapolation.

#### Step 2: Training-data real-world goals to mathematical goals

The original RCT papers and the later meta-analysis all required a statistical model and choice of inference procedure. Economists often choose linear models for their interpretability. We chose a hierarchical linear model to allow partial pooling across the different studies, reflecting both their shared information and idiosyncrasies. Moreover, we took a Bayesian approach and provided a prior as part of the model.

We reported Bayesian posterior summaries such as posterior means and variances. Given a hierarchical model and nonconjugate priors, these summaries must be approximated. So the mathematical goal is to report accurate approximations of these quantities for the particular chosen model.

##### Trust

Trust could break down in this step because we know that models are essentially always misspecified in practice. Linear models of conditional mean dependence are highly interpretable but cannot capture nonlinear trends that may exist (and can be captured by other models). Even if the policy intervention is represented by a binary variable, linearity in raw household outcomes is incompatible with linearity in log space, and the distinction matters for extrapolating to new settings. Focusing on the mean itself is an influential choice; the mean effect of microcredit may be positive even if just a small portion of people actually benefit and even if some are harmed. In contrast, what we might really wish to understand is whether many people benefit from microcredit or the community as a whole experiences net benefit. Moreover, reporting a posterior mean and variance alone, without additional visualizations, can hide posterior multimodality, heavy tails, or other distributional features that might cause us to pause and dig deeper into an analysis.

#### Step 3: Mathematical goals to algorithms

We chose to approximate the Bayesian posterior mean and variance summaries using Hamiltonian Monte Carlo (HMC) (*15*). In related work (*16*), we have considered variational Bayesian (VB) approximations of these posterior quantities instead; in the latter case, we used mean-field VB approximations (*17*) and linear response corrections (*18*, *19*).

##### Trust

Trust could break down in this step if the algorithm did not accomplish the mathematical goal. For instance, Markov chain Monte Carlo (MCMC) estimates of posterior expectations will converge to their exact values when an algorithm is run for enough time. However, despite a large literature on mixing rates, there are typically no concrete, computable bounds on performance after running a particular MCMC algorithm for a particular finite time. Analogously, VB will return a match to the exact posterior when used with a sufficiently large family of distributions and enough computation time. But in practice, the family of approximating distributions is typically limited, and the Kullback-Leibler (KL) divergence between the approximating and true posterior will be strictly greater than zero. Even moderate KL divergence values can correspond to arbitrarily large discrepancies between exact and reported posterior means and variances (*20*). While there is work on using more expressive families of distributions in VB, these face practical challenges and typically lack computable guarantees on quality.

#### Step 4: Algorithms to code

As increasingly complex methods are used for data analysis, a practitioner will often turn to existing software packages. Here, we used Stan for HMC (*21*). Giordano *et al.* (*16*) developed new code for VB.

##### Trust

Trust could break down in this step if there are bugs in the code. There is robust software engineering guidance on how to test code, including unit testing. Data analysts often rely on a software package for implementation and thus, to some extent, often outsource much (but certainly not all) of the establishment of trust in this step. Fitting the model in Stan, a well-tested probabilistic programming language, gave us confidence in the inferential part of our computation. However, there is still the entire data and analysis pipeline to consider. Recall the infamous Excel error in (*22*); see (*23*) and (*24*).

### Case study: Election forecasting

We similarly walk through the parts of our taxonomy, and how they relate to trust, with an example from our development of a public poll-aggregation algorithm for election forecasting.

#### Future real-world goals

In spring 2020, one of us collaborated with a political science graduate student and a data journalist at the *Economist* magazine to produce an ongoing state-by-state and national-level forecast of the upcoming U.S. presidential election (*4*, *5*). The forecast automatically updated as new polls arose during the campaign.

#### Step 1: Future real-world goals to training-data real-world goals

A particular challenge of this problem is incorporating diverse sources of data: national polls, state polls, economic statistics, and past state and national election results, as well as knowledge reflecting political science understanding of day-to-day changes in voting behavior and survey responses.

##### Trust

By analogy to the microcredit example, trust could break down in this step if the data used in the model were deemed incomplete in the sense that there are other available data that could be used to noticeably improve the forecast. For example, a forecast based entirely on national polls would have problems staying up to date with the latest state poll results. Particularly relevant to the 2016 and 2020 elections were concerns about poll bias due to nonresponse; for instance, we might be concerned that fewer Republicans responded to polls, especially in key swing states.

#### Step 2: Training-data real-world goals to mathematical goals

Constructing the model was more difficult than you might think. The probability distributions representing the underlying public opinion and survey data had to serve as a bridge connecting poll and economic data, political science understanding, and statistical structure (sampling error, nonsampling error, and variation of opinion over time in the 50 states).

Concretely, we started with an existing forecasting model in political science (*25*), which in turn was based on earlier models for poll aggregation and election forecasting. The unique feature of our approach compared to other forecasts for the 2020 election was fitting a single model combining information from national polls, state polls, and economic and political fundamentals based on previous election outcomes.

Challenges in setting up this model included the following. We wanted to account for nonsampling error at both the state and national levels, including possible systematic biases favoring one party or the other; our error terms did not correct for biases, but they should allow the model to express forecasts with appropriate uncertainty. We aimed to capture a time series of opinion changes during the campaign. We wanted to interpret the historical predictive power of economic fundamentals in the context of increasing political polarization. In addition, we aimed to acknowledge unique features of the 2020 campaign. A particular statistical challenge arose in modeling the correlations of polling errors and opinion trends across states; there was nothing like the amount of data available to estimate a 50 × 50 covariance matrix from any purely empirical procedure, so we knew that strong assumptions would be necessary.

##### Trust

Trust could break down in this step if any of these strong assumptions or modeling choices could be replaced with other reasonable choices and lead us to substantively different conclusions. Some of our initial modeling choices regarding between-state correlations were flawed, as we noticed after seeing some predictive intervals that seemed implausible. Unfortunately, we noticed some of these errors only after the forecast went live, and we were forced to reboot our model and publicly explain our changes.

#### Step 3: Mathematical goals to algorithms

As with the microcredit case study, we used HMC to approximate and summarize the Bayesian posterior distribution on election outcomes and other posterior quantities of interest for political understanding. The dataset and model were small enough and our time scale was gentle enough (requiring updates every day, not every hour or minute) that there was no need to use a fast shortcut algorithm.

##### Trust

As discussed in the previous example, trust could break down if the approximation quality were poor.

#### Step 4: Algorithms to code

We used R and Stan for HMC; we also compiled and cleaned survey and election data.

##### Trust

Our general experience with Stan allowed us to trust its computation, so the main concerns with the code were in the R scripts we wrote to prepare the data, set up covariance matrices, and postprocess the inferences.

## BUILDING TRUST

Above, we have seen a number of ways that trust can break down across the taxonomy. But crucially, there are also many ways for researchers to build trust. We next place existing work into our taxonomy. We start from the bottom of Fig. 1 and work our way up. In particular, the italicized vertical connectors in Fig. 1 give terms related to trust for the corresponding step. The horizontal connectors show terms that describe matching between goals and outputs at this level.

While we hope to highlight ways that analysts and researchers can grow trust, we do not intend to suggest the existence of a perfect analysis or a foolproof solution—just as we might improve software engineering through testing, but we do not expect even the most careful testing to entirely eliminate bugs. Likewise, we try to distinguish between tools that are available to most analysts and tools that would require an unusually large monetary expense, time budget, compute cost, or other resources.

### Algorithms and code

#### Fidelity

Trusting code to faithfully represent an algorithm ("fidelity") is sometimes considered prosaic and perhaps even outside the purview of the data scientist, especially when using standard software packages. Nonetheless, this step forms the bedrock of the analysis; if trust breaks down here, trust in the entire analysis will fail. Indeed, bugs in data analyses have led to unsupported justification for treatment of patients in clinical trials, as discussed in (*26*), and influential but unsupported economic policy advice, as discussed in (*23*) and (*24*).

#### Reproducibility

The name of the core issue here varies widely across the literature. In the present paper, we follow the usage of Conclusion 3-1 of the National Academies report on “Reproducibility and Replicability in Science” (*27*) and label an analysis as "reproducible" if identical results are achieved when the same data are analyzed again with the same code. Despite this definition seeming somewhat limited, many data analyses (including some of our own in busy research projects) do not meet this standard. For instance, some data analyses do not provide code. Some do not even describe their algorithm in enough detail for the reader to produce equivalent code (*28*). Moreover, unequal computing resources can form a barrier to actually checking reproducibility in practice; some modern analyses require resources enjoyed only by a handful of large companies (*29*). Even with full descriptions and code and adequate resources, checking reproducibility can be challenging and time-consuming. For instance, the rapid advances of the ML field give rise to continually changing software packages and dependencies; even when the precise set of dependencies used by a project is known, setting up the same ecosystem can be burdensome. In addition, probabilistic methods often take advantage of randomness; without access to the random seed, two separate runs of the same code in the same environment may yield different results.

#### Supporting reproducibility

Nonetheless, these observations suggest clear ways to build trust at this step, via open community engagement, code sharing, and data sharing. Even when using standard software packages, analysts typically write their own code wrappers to call these packages for the specific data and model at hand, and this new code needs to be checked. Heil *et al.* (*30*) propose a gold standard where data analysts set up a single command that exactly reproduces the full analysis and a silver standard that requires easy access to package dependencies and any random seeds. Modern computational tools that manage models (*31*) and aid reproducibility (*32*) can help researchers achieve the gold standard. It might be easy to overlook code that goes into data preprocessing, but this code is part of an analysis pipeline too; for instance, NASA failed to detect ozone reduction for much of the 1970s and 1980s (despite having appropriate sensors) due to an unexpected side effect of data preprocessing (*33*). Gebru *et al.* (*34*) propose “datasheets for datasets” to fully detail data provenance, including any processing that may have gone into their creation. Permissive open licensing at every step of the pipeline can encourage reproducibility and proper attribution (*35*, *36*).

#### Case study

During the election forecasting case study above, we opened up the process and results to a wide audience. Publication and daily updates in the *Economist* meant that thousands of readers would see each forecast. We made data and code publicly available and conveniently accessible (in this case, on GitHub). This ready access allowed outsiders to download the code, run our analysis themselves, and explore places where something could be going wrong. Outside readers did find problems, which we were able to track down to bugs and conceptual problems with our model. In addition, the sense of public responsibility motivated us to check carefully when forecasts did not look right.

### Mathematical goals

#### Theoretical guarantees

At this level, we want to understand whether our algorithm achieves our stated mathematical goals. Often research will abstract the algorithm away from the code; for example, a researcher might establish theoretical guarantees on how well a particular algorithm can be expected to achieve a goal such as accuracy or compute time. These guarantees can help build trust. However, they typically cannot be entirely relied upon to ensure accuracy. The exact set of assumptions needed for the guarantee may not hold in practice or may not be possible to verify exactly in practice. A large amount of research concentrates at this level. For instance, it is standard for any paper addressing Bayesian posterior approximation to come supported by theory. We likely see this abundance of research since the question of whether an algorithm achieves a precise mathematical goal has a relatively objective answer.

#### Checking the algorithm via the code

Practical checks at this level can examine the actual instantiation of the algorithm in code. Analysts might check the performance of the algorithm-code pair on representative problems or provide post hoc checks on the results of the algorithm-code pair when run on the dataset at hand. For instance, as we have seen in the case studies, a sufficiently complex Bayesian analysis typically aims to approximate summaries of the posterior. Geweke (*37*), Cook *et al.* (*38*), and Talts *et al.* (*39*) present simulation-based methods for checking the accuracy of a Bayesian approximation algorithm (via its implemented code). We and others have also developed methods for checking approximate computation on a particular dataset of interest; e.g., Yao *et al.* (*40*) and Huggins *et al.* (*20*) have presented methods for evaluating variational inference. These new approaches can help to identify mismatch between the mathematical goals and the joint implementation of the algorithm and code.

#### Case study

In the election forecasting case study, we simulated fake polling data and then saw how well the fitting procedure could recover the assumed parameters and underlying time series. The check proceeded as follows: (i) set hyperparameters of the chosen model to fixed, reasonable values; (ii) simulate time series of national and state public opinion using the generative time series including the between-state correlation matrix assumed under the model; (iii) simulate national and state polls at the same sample sizes and dates (in days before the election) as in the 2016 campaign but with data generated according to the simulated underlying time series plus error, including both sampling and nonsampling error; and (iv) fit the model to these simulated data. It is no surprise that we should be able to roughly recover the hyperparameters and time series, but given the complexity of the model, it is useful to check and also to get a sense of the precision of the recovery.

### Training data and real-world goals

To build trust at this level, we want to ensure that our mathematical goals are meeting our real-world data analysis goals, at least on our training data. Building trust here presents a new challenge relative to previous levels; in particular, we must now ask what it means to perform well on real-world goals. The answer to this question is necessarily more open to multiple perspectives and context dependent than the mathematical problem an analyst reduces this question to in step 2. Indeed, analysts often reduce the more abstract and ill-defined real-world problem to a standard mathematical problem not only to apply familiar data analysis tools but also to avoid claims that the analyst is lacking objectivity; see table 1 in (*41*) and the surrounding text for a discussion of how multiple perspectives and context dependence relate to descriptions of subjectivity and objectivity in an analysis. We emphasize the distinction in character between this level and previous levels with the color of connectors in Fig. 1.

The challenge of this level need not be due to communication difficulties between a data analyst and a domain expert. In both of our case studies, a single person is both a data analyst and a domain expert. Yet there remain fundamental challenges.

#### Proxy measures to assess performance

We often choose proxy measures to assess performance of a method, so we might wonder how stable our results are to these choices. We may choose proxies when questions in the social sciences, sciences, and engineering are difficult to operationalize directly or when these questions might require substantial new modeling and inference development. For example, we might use a mean effect to understand the efficacy of microcredit because a mean is straightforward and standard to capture via a linear model, even though it might be more appropriate to judge the effect of microcredit by the proportion of people to whom it is beneficial. As another example, many methods default to prediction with simple losses when the real-life goal or loss is difficult to quantify. For instance, 0-1 loss is a common choice of convenience in classification problems, but real-life classification loss is typically both unbalanced and difficult to quantify precisely. Prediction is generally an easy (statistical or mathematical) problem precisely because it is defined in terms of a clear ground truth that we can compare our predictions to. However, even problems that are naturally framed as prediction, such as medical diagnosis, may be mistranslated into a concrete optimization problem; e.g., consider a model for detecting cancerous skin lesions that inadvertently trains on surgical ink markings (*42*).

These proxy measures of performance form part of a larger phenomenon in scientific research. Hard questions often get translated into questions that are easier to answer but possibly substantively further from the question we want to ask. At its core, this critique is related to the question of how problem formulation can affect scientific outcomes (*43*). Relatedly, it is important to consider whether we care about prediction or explanation (*44*). A particular analyst’s real-world goals might be best addressed by optimizing a black box that makes highly accurate predictions. A different analyst might be best served by constructing a more interpretable model with strong explanatory power. A third analyst might best do something in between.

Proxy measures of performance can have fairness implications. For instance, an economic intervention can have positive average effects while benefiting only a small part of the population that is already well off, or medical interventions tested only on men might expose women to unknown risks. See (*45*–*47*) for further discussion of fairness in ML, currently an active research area.

#### Stability to model choice

Just as we might choose proxy measures in part out of convenience, we might choose particular models to use in part out of convenience. In this case, we might ask about the stability of our decisions under different reasonable modeling choices. Yu (*48*, *49*) has advocated for the importance of stability of many forms, including stability under model choice, in data analysis.

Different representations or summaries of our data could also be seen as part of the choice of model, and we are interested in stability across reasonable choices of summary. As an example, consider choosing among competing network embeddings. Ward *et al.* (*50*) examine this problem within the framework of Yu and Kumbier’s (*51*) principles for veridical data science: predicability, computability, and stability. For some problems, we may be interested in embeddings that preserve particular features of the observed network or in embeddings that translate to better performance for some downstream task (e.g., link prediction), rather than in estimating a true underlying embedding or even mere features of that embedding, such as the manifold in which those embeddings lie, as in (*52*, *53*).

#### Testing stability to model choice

To test stability to these various choices in practice, Silberzahn *et al.* (*54*) document variations among 29 distinct research teams’ analyses of a shared real-world goal, equipped with shared training data: whether soccer referees are more likely to give red cards to players with dark skin tones. Variations in adopted analytic strategies ranged in breadth from differences in which predictors are included to how dependent observations are (or are not) adjusted for to statistical modeling assumptions (e.g., linear or logistic regression).

#### Lower-cost tests of stability or robustness to model choice

Convening multiple research teams for a problem is not always feasible, at least not at first, due to budget and time costs. So we might wish to have lower-cost checks usable by a single team. For instance, in (*55*), we construct prediction scores as a measure of agreement between data-generating mechanisms (e.g., comparing preregistration or pilot data to realized experimental data). We show that modifications to the predictive model (used to construct the predictions) adjust the lens through which the prediction scores can pick up on varying types of differences between the underlying data generating mechanisms. This check goes beyond the mere reproduction of numerical outputs but more broadly accounts for how the real world interacts with the inferential chain: how the real world goals are interpreted and formulated as mathematical goals.

Another body of literature looks at quantifying the robustness of the conclusions of a data analysis to likelihood and prior choice in a Bayesian analysis (*56*), with tools to interface with MCMC (*57*) and variational methods (*19*, *58*). A tricky aspect of operationalizing this work is deciding how to mathematically capture a range of reasonable models; the easiest option is to vary a hyperparameter of a model (that may control the model parametrically or nonparametrically) over some range that the analyst deems reasonable. Other options include visualizations to aid analysts and domain experts in making appropriate robustness checks (*59*).

Even the lower-cost tests, though, typically require additional thought and computation beyond the initial analysis. Moreover, even the most inexpensive and automated robustness checks are often not yet part of standard data analysis software or generally an expected part of a data analysis. Perhaps due both to the challenge of subjectivity at this level and the additional effort required, we often do not see checks at this level in individual data analyses.

### Future real-world goals

At this level, we want to build trust that the results from our particular data analysis are relevant to conclusions and decisions we will make at other places or times.

#### Replicability and robustness to data variation

Again, building on Conclusion 3-1 in (*27*), we say that an analysis is "replicable" if similar results are achieved when the study is repeated with fresh data. A replicable study does not strictly require reproducibility (or matching of goals and outputs at other levels of Fig. 1). However, it is hard to imagine how a nonreproducible analysis could be successfully replicated.

We can still speak of stability and robustness but now to changing data rather than changing model choices. For instance, ultimately, we wish to apply the conclusions of our microcredit case study to decide whether to pursue microloans in new places in the future or in the same places but at necessarily different (future) times. If we concluded that microcredit alleviated poverty based on our original data, can we continue to trust these conclusions?

#### Testing replicability

As in the previous case, the best option for testing replicability is to run the experiment many times in many different conditions. The Open Science Collaboration (*60*) brought replicability to the forefront of the scientific discussion via an attempt to replicate 100 high-impact psychological studies. To the extent that teams are able to run full analyses that may shed light on future use cases, Mitchell *et al.* (*61*) propose “model cards” to document model details, training data, evaluation data, and evaluation metrics. However, running a data analysis many times under different circumstances may be costly, and doing so does not eliminate the need to make future policy decisions using past analyses. To that end, there is a need for automated tools that can assess sensitivity and robustness to data changes that might reasonably reflect changes we expect to see before applying policy.

#### Data resampling schemes

Classical tools such as cross-validation and the bootstrap can provide a notion of sensitivity by rerunning a data analysis many times. Likewise, when many predictions and a ground truth are available, researchers can check whether their uncertainty forecasts are calibrated; that is, one can check whether the proportion of times an event was predicted agrees with the forecasted uncertainty. However, these checks rely on an implicit assumption that the data-generating distribution of future data is fundamentally the same as that of the data used for the checks. This assumption is often at least somewhat inappropriate. Recall that we hope to capture and apply universal truths from a data analysis. We therefore might not apply decisions from our data analysis to a wildly different context; nonetheless, we still might expect and wish to anticipate small but substantive changes across datasets of interest. D’Amour *et al.* (*62*) have found that many conclusions of AI analyses do not hold up when used in real life despite being trained and tested in standard ML pipelines. In our own work, we reasonably expect regional differences in the effects of microcredit and differences across time in elections. One option is to use cross-validation variants that respect temporal or spatial structure (*63*), but even these variants presuppose that future data fit a past trend or symmetry.

#### Stress tests

D’Amour *et al.* (*62*) suggest stress-testing data analyses to explore performance in practically relevant dimensions and to attempt to identify potential inductive biases. Ribeiro *et al.* (*64*) also suggest running methods on a variety of meaningful datasets to diagnose different types of hidden issues. A challenge is finding appropriate datasets. One option that we have developed and applied to our microcredit case study is assessing sensitivity of an analysis to dropping a small amount of data (*65*). If an analysis is driven by a small proportion of its data, then we might not expect it to generalize well to other scenarios. In theory, this sensitivity can be checked directly by rerunning the data analysis with every small proportion of data removed; in practice, this naive approach is astronomically costly on even small datasets. So we develop an approximation that can be checked directly. In particular, when our approximation detects sensitivity, it returns the problematic small subset of data. So the analyst can drop this small data subset and rerun the analysis at the cost of just one additional data analysis.

#### Generalizability versus security

The methods above address concerns about the "generalizability" of an analysis; populations may subtly differ across space or time, and these changes may in turn affect the efficacy of, e.g., an economic or medical intervention. A distinct and substantial literature studies "adversarial examples" generated by a nefarious actor and methods to counter them (*66*). We emphasize that one may be concerned about generalizability even when no security concerns or adversaries are present.

#### Indirect checks

The checks discussed so far at this level try to assess generalizability directly. Analysts may call on other forms of evidence, though, to try to understand whether decisions may generalize well from the particular training data at hand. One form of evidence is "explainability." If we know that predictions of cancerous skin lesions are based on surgical ink markings (*42*), then we might expect that the method will generalize poorly to cases where experts have not already effectively made the predictions and marked them for us. By contrast, if the method’s predictions have a convincing medical explanation, we may be more likely to trust the method to generalize well to new image sources. We direct the interested reader to a wide, and actively expanding, literature on explainability in AI methods, a full accounting of which is outside the scope of the present work; see, e.g., (*67*–*71*).

Another indirect check is to assess whether the model is able to capture salient aspects of the data that are not directly part of reported summaries. Such Bayesian "model checking" originated with (*72*) and (*73*); this research arose in an era when computations were relatively simple, so the focus was on fit of model to data. In modern work, Bayesian model checking often assesses whether the joint model-algorithm-code mechanism does a good job of describing the available training data, sometimes with additional granularity (*74*).

#### Case study: Microcredit efficacy

We applied exactly the dropping data check described above to our analysis of microcredit in (*75*). We hoped that various features of the analysis would decrease sensitivity: hierarchical Bayesian sharing of power across datasets, regularization from priors, and a tailored likelihood meant to more carefully reflect the data-generating process. However, we still found that the average effects were sensitive to dropping less than 1% of the data, but the estimated variance in treatment effects across studies was more robust (*65*).

#### Case study: Election forecasting

The election forecasting case study used sense checks related to the ideas above to assess whether its predictions could be trusted to generalize to the 2020 election. Some months elapsed from the start of our modeling process and the *Economist*’s launch of the forecast. This lag did not come from coding (as code is straightforward in Stan) or from running the code (the dataset including all polls was small enough that even without any real efforts at optimization, the code ran in minutes) but rather from the steps of building and checking the model. Once we had code that compiled and ran, we fit it to data from 2008, 2012, and 2016; these were earlier elections where we had a large supply of state and national polls, so we could mimic the 2020 forecasting we planned to do. Various early versions of our model produced results that did not make sense; for example, time trends seemed to vary too much from state to state, which implied that some variance parameter in our model was too large so that these time series were insufficiently constrained. This checking was valuable for its own sake and also because, to do so, we needed to design graphs whose forms we would use when plotting models fit to the 2020 campaign data.

**Fig. 2.**
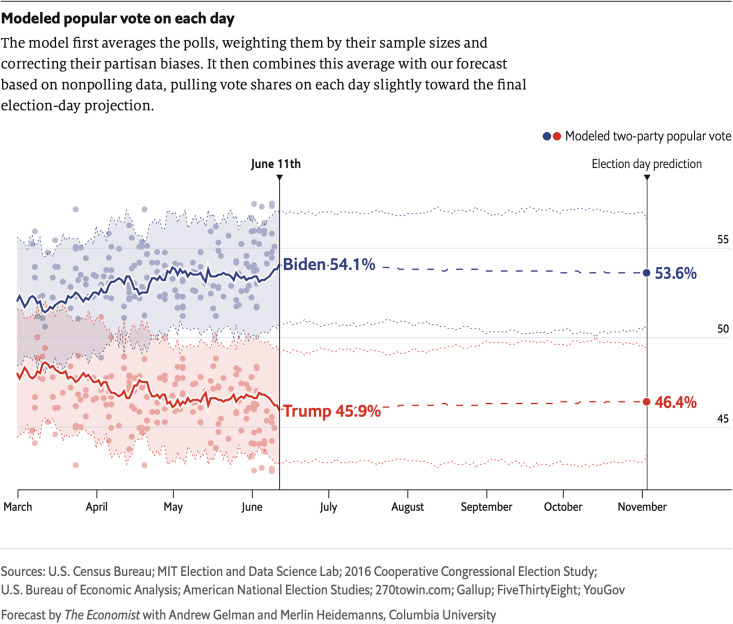
Our election forecast for the *Economist* on the day of its release in June 2020. Before taking this forecast public, we went through a series of checks of the data, model, and fitting procedure. We made further changes to the model as the campaign went on.

Before releasing our predictions to the public in early June, 2020, we fit the model to the data available up to that point and checked that the inferences and our summary of them (see Fig. 2) seemed reasonable, in the sense that they were consistent with our general understanding of the campaign and election. Any model is only as good as the data it includes, so even if the fit has no statistical errors, we would be concerned if it produced forecasts that were far off from our general beliefs. We also examined the state-by-state forecasts from a competing model produced by the Fivethirtyeight.com team and found some implausible predictions, which we attributed to issues in how their model handled between-state correlations (*76*).

During the summer and fall of 2020, we continued to monitor our forecast. In particular, we were concerned that, months before election day, the estimated probability of Biden winning the electoral college was over 90%, which did not seem to fully capture our uncertainties. We looked carefully at our code and performed more simulations, refitting our model under alternative scenarios such as removing polls from one state at a time, and eventually we found some bugs in our code and other places where we were unsatisfied with the model, most notably in our expression of the between-state correlation matrix. After a couple weeks of testing, we released an improved model along with a correction note on the *Economist* site.

Finally, we compared to the actual election outcomes. We found that our model performed well but not perfectly. The popular and electoral vote margins fell within our 90% forecast intervals, with 48 of 50 states predicted correctly. However, the election was closer than our point forecast, with Biden performing consistently worse than predicted in almost every state. Polls were off by about 2.5 percentage points in two-party vote share across the country; our forecast did not anticipate this error, but it was included as a possibility in the correlated polling error model. Including a correlated error term allowed the forecasts to acknowledge nonsampling error but is no substitute for predicting the direction of the bias.

## DISCUSSION

Perfect accuracy is not a requirement for trust. We are able to trust many of the systems that we interact with in our day-to-day lives despite their uncertain predictions. Weather predictions are not always correct, and yet they are still useful; many people are injured or die in car crashes, and yet we rely on cars to travel from place to place; we do not expect doctors to be able to comprehensively restore us to our previous health, and yet we trust our doctors. Even for probability models, our thresholds for acceptable accuracy may depend on the setting or type of model. For example, much of classical statistics developed out of agricultural settings. As a result, many statistical models are well aligned with the physical sciences and essentially act as deterministic or mechanistic process models with noise. In these models, the error is often treated like a nuisance parameter and included because we would not be able to estimate the model otherwise. Other statistical models are more descriptive and are fundamentally models of variability, such as in economics or the social sciences. A third class of models is even more disconnected from observable physical processes; these discovery or exploratory models are specifically designed to capture fuzziness. Examples include clustering models, topic models, and other unsupervised learning approaches. We naturally expect different levels of accuracy for models in each of these classes and, especially for discovery or exploratory models, defining accuracy can be more difficult.

In our work, we have focused on fostering trust in a Bayesian context in a way different from the traditional notion of estimating the posterior probability that a model is true. Rather, we recognize that our models are assumptions, and trust relates in part to the robustness of our results and subsequent decisions to many types of explicit and implicit assumptions. There is also a large non-Bayesian literature on evaluating statistical and ML methods via predictive performance [e.g., (*77*–*79*)]. These approaches have links to Bayesian predictive inference (*80*), just as there are links between deterministic and probabilistic models (*81*). However, we have found in applied work that real-world model improvements do not always show up as clear increases in prediction accuracy (*82*); this seeming discrepancy makes sense because a key goal in many applications is to make decisions that apply outside the scope of training data.

More generally, we highlight that trust itself is not binary. We have here laid out a variety of points in an analysis where trust can fail or be increased, but even at each point, trust lies on a spectrum. Heil *et al.* (*30*) note that, even at the level of code reproducibility, there lies a spectrum of trust: from not providing code or details to providing them but without easy documentation or use all the way to providing well-documented code that duplicates an existing analysis with a single command. We analogously argue in (*55*) for treating trust as a spectrum, rather than a binary, at other levels of our present taxonomy. In almost all settings, there will be some variation (due to different operationalizations of the real world goals, to stochastic elements in the algorithm, or even to random number generation in the code), even if only slight. Instead, we should focus on quantifying how much variation is present. In (*19*), we make the distinction between sensitivity and robustness as follows. Sensitivity measures the (continuous or near-continuous) degree of change in some reported value due to, e.g., changing the model. That is, sensitivity is a well-defined continuous quantity. However, robustness is a subjective judgment call based on sensitivity; we say an analysis is nonrobust if a particular observed change is deemed important in that a change in reported value affects a particular actionable decision. We see a version of this distinction in our check for dropping data (*7*) as follows. We assess sensitivity by computing the biggest change in our reported quantity of interest across different sizes of dropped data subsets. Ultimately, an analyst must decide if robustness is a concern by asking whether the change in data required to see a substantively different conclusion is too small.
